# Exploiting Electrode Nanoconfinement to Investigate the Catalytic Properties of Isocitrate Dehydrogenase (IDH1) and a Cancer-Associated Variant

**DOI:** 10.1021/acs.jpclett.1c01517

**Published:** 2021-06-25

**Authors:** Ryan A. Herold, Raphael Reinbold, Clare F. Megarity, Martine I. Abboud, Christopher J. Schofield, Fraser A. Armstrong

**Affiliations:** Department of Chemistry, University of Oxford, Oxford OX1 3QR, United Kingdom

## Abstract

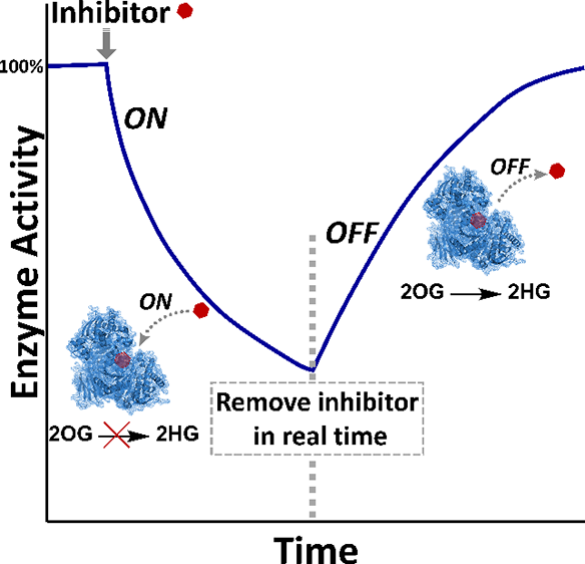

Human isocitrate dehydrogenase (IDH1) and its cancer-associated variant (IDH1 R132H) are rendered electroactive through coconfinement with a rapid NADP(H) recycling enzyme (ferredoxin-NADP^+^ reductase) in nanopores formed within an indium tin oxide electrode. Efficient coupling to localized NADP(H) enables IDH activity to be energized, controlled, and monitored in real time, leading directly to a thermodynamic redox landscape for accumulation of the oncometabolite, 2-hydroxyglutarate, that would occur in biological environments when the R132H variant is present. The technique enables time-resolved, in situ measurements of the kinetics of binding and dissociation of inhibitory drugs.

Genes encoding isocitrate dehydrogenases (IDH) are the most frequently mutated metabolic genes associated with cancer.^1,2^ Wild-type IDH1 is a homodimeric cytoplasm-localized enzyme requiring Mg^2+^, which catalyzes the reversible oxidative decarboxylation of isocitrate to 2-oxoglutarate (2OG) using NADP^+^.^3^ Active site substitutions, frequently at arginine-132, switch the dominant IDH reaction from isocitrate oxidation to 2OG reduction, producing (R)-2-hydroxyglutarate (2HG).^4,5^ These neomorphic mutations cause 2HG—normally present at very low levels—to accumulate in cells,^4^ inhibiting enzymes including 2OG/Fe(II)-dependent oxygenases and disrupting vital processes such as DNA repair and histone and DNA demethylation.^6,7^ The IDH1 R132 variants are linked to numerous cancers, and 2HG is characterized as an “oncometabolite”.^3,8^ Notably, IDH1 R132H is associated with >80% of grade II/III gliomas and is a common mutant allele associated with acute myeloid leukemia (AML).^3,9−11^ To treat IDH1-associated cancers, small molecule drugs inhibiting IDH1 variants have been developed: Ivosidenib (AG-120) is FDA-approved to treat AML,^12^ and other inhibitors are in clinical trials.^2,7,13^ Here we report a conceptually new approach to characterizing IDH variants that should also be widely applicable to other NAD(P)(H)-dependent enzymes, a class which constitutes one-sixth of all enzymes.^14^ Despite not using long-range electron transfer, these redox enzymes are now rendered electroactive through a versatile new physical platform called the “Electrochemical Leaf”.

The Electrochemical Leaf is a nanoporous metal oxide electrode loaded with an electroactive enzyme cascade enabling it to drive rapid localized nicotinamide cofactor (NADP(H)) recycling, making it possible to energize, control, and observe the action of these enzymes directly by dynamic electrochemical methods.^15−23^ When trapped in the nanopores of an indium tin oxide (ITO) electrode alongside electroactive ferredoxin-NADP^+^ reductase (FNR), NADP(H) dehydrogenases such as IDH (E2) are likewise rendered electroactive via efficient, highly localized recycling of NADP(H) (Scheme 1A).

**Scheme 1:**
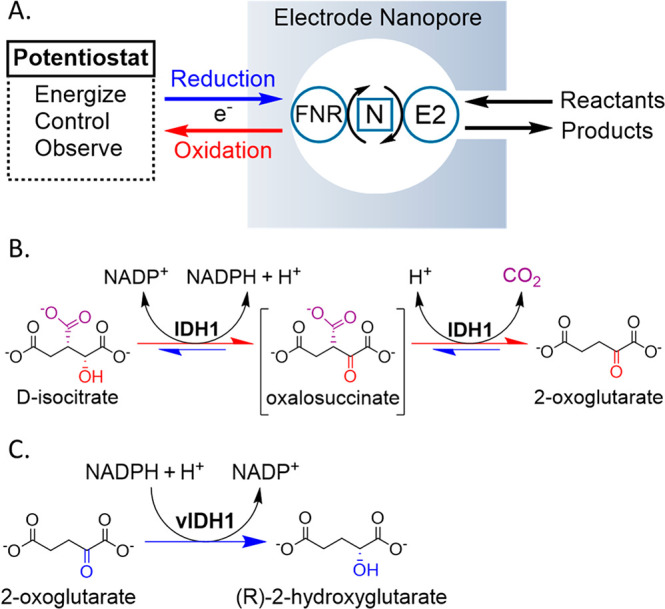
“Electrochemical Leaf” and IDH1 Reactions (A) Principle of the “Electrochemical Leaf” showing the minimum functional unit required to energize, control, and observe the real-time activity of an NADPH dehydrogenase: FNR, E2 = NADP(H) dehydrogenase, N = nicotinamide cofactor (NADP(H)). (B) The reaction (isocitrate oxidation and its reverse process) catalyzed by wild-type IDH1; oxalosuccinate is an enzyme-bound intermediate. (C) “Gain of function” reaction (unidirectional reduction of 2OG to 2HG) catalyzed by IDH1 variants (vIDH1), including IDH1 R132H.

In generic terms, the electrode is denoted by (FNR+E2)@ITO/support, where E2 is an NADP(H) dehydrogenase. The typical support is pyrolytic graphite “edge” (PGE) or titanium foil; a layer of ITO nanoparticles (<50 nm) is electrophoretically deposited to 1–3 μm depth. The enzymes (FNR and E2) are loaded into the pores by dropcasting a premixed solution and then rinsing thoroughly with buffer (see the Supporting Information).

The minimum functional unit is the {FNR, NADP(H), E2} molecular triad, and output rates are optimized by adjusting component ratios. The enzymes are concentrated in the electrode nanopores: the amount of FNR can be quantified by integrating the FNR-bound FAD redox signal (see the Supporting Information), which at pH 8 is clearly visible at a potential of −0.38 V vs SHE, notably much more negative than that of unbound FAD (−0.25 V).^15^ A typical FNR loading at pH 8 corresponds to 0.7 mM in a 1-μm-deep layer, ignoring the volume due to ITO material.^22^ As controlled via an electrochemical workstation, FNR acts as the transducer, interconverting electrons and NADP(H) with high efficiency and reversibility in the nanoconfined environment.^16^ The catalytic rate in either direction, reduction or oxidation, is displayed directly on a computer screen as current. Where needed, mass transport of reagents to and from the electrode can be assisted by rotating the electrode.

The activities of homodimeric (2 × 47 kDa) IDH1 and IDH1 R132H (hereafter R132H) for their respective metabolite interconversions, isocitrate/2OG, CO_2_, and 2OG/2HG, are shown in Figure 1. The cyclic voltammograms (CVs) give a direct read-out of how rates (current) depend on free energy (as potential). Electrodes containing nanoconfined FNR and IDH1 or R132H were prepared as described in the Supporting Information. The black traces show the cyclic voltammetry for quasi-reversible FNR-catalyzed NADP^+^/NADPH reduction and oxidation at a stationary electrode measured before injecting the IDH substrate. When coupled to local NADP(H) recycling, IDH1 (Figure 1A, B) is an excellent catalyst for isocitrate oxidation; the steep potential dependence of the oxidation current shows that the rate is determined by how fast FNR can transfer electrons to recycle local NADP^+^ for reuse by IDH1 (FNR and IDH1 dimers were loaded in a 2:1 ratio). By contrast, and consistent with standard solution kinetics,^24^ R132H-catalyzed 2OG reduction (Figure 1C, D) is much slower, manifesting low currents and persistent sigmoidal CVs that are characteristic of limitation by R132H, despite the electrode being loaded with a R132H/FNR ratio 4-fold higher than the equivalent IDH1 electrode. The lower catalytic activity of R132H results in a relatively larger background due to electrode capacitance.

**Figure 1:**
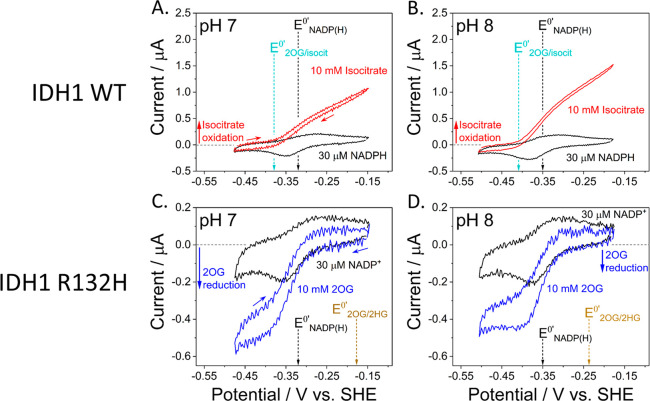
Cyclic voltammograms for nanoconfined IDH1 (A, B) and IDH1 R132H (C, D) catalysis at pH 7 and 8 (for pH 6 and 9, see the Supporting Information). Conditions: stationary (FNR+E2)@ITO/PGE electrode, electrode area 0.03 cm^2^, scan rate 1 mV/s, temperature 25 °C, 60 mM mixed buffer (20 mM each: MES, TAPS, CHES), 10 mM MgCl_2_, 30 μM NADP(H), 10 mM substrate (isocitrate or 2OG), volume: 4 mL. Enzyme loading ratios (molar): FNR/IDH1; 1/0.5 and FNR/R132H; 1/2. *E*^0′^_NADP(H)_, *E*^0′^_2OG/isocit_, and *E*^0′^_2OG/2HG_ denote formal potentials for NADP^+^/NADPH, 2OG/isocitrate, and 2OG/2HG couples (see Figure 3).

Further experiments were carried out to test the bidirectionality of catalysis by IDH1 and R132H. For IDH1-catalyzed 2OG/isocitrate interconversion (Figure 2A), the CO_2_ that is required was produced catalytically in situ from bicarbonate present at 0.1 M in the buffer using carbonic anhydrase (CA), a highly active Zn-enzyme, that was coentrapped in the electrode nanopores;^22^ notably a 50:1 2OG/isocitrate ratio was needed to equalize the oxidation and reduction currents. A formal reduction potential for the 2OG/isocitrate interconversion (*E*^0′^_2OG/isocit_) of −0.40 V vs SHE at pH 7.7 at the local CO_2_ level was calculated from the Nernst equation using the clearly defined zero-current potential (*E*_eq_) obtained with the 50/1 ratio.

**Figure 2:**
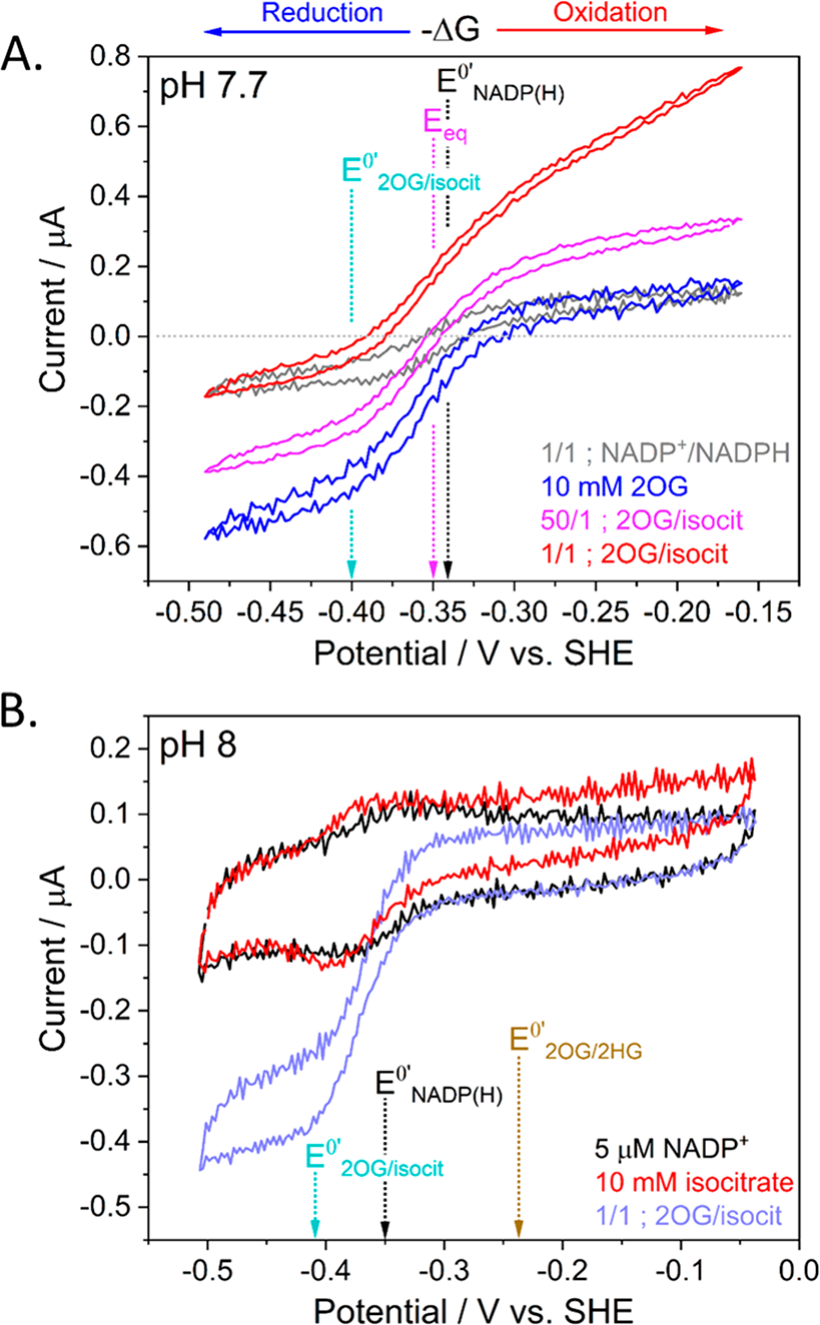
(A) Cyclic voltammetry measuring bidirectionality of IDH1-catalyzed 2OG/isocitrate interconversion at different 2OG/isocitrate ratios. (B) Cyclic voltammetry measuring the ability of R132H to catalyze isocitrate oxidation. Conditions (A and B): electrode area 0.03 cm^2^, scan rate 1 mV/s, temperature 25 °C, volume 4 mL, 10 mM MgCl_2_. (A) (FNR+IDH1+CA)@ITO/PGE electrode, 15 μM NADP^+^, 15 μM NADPH; HEPES and NaHCO_3_ at 0.10 M; rotated at 1000 rpm. Magenta trace: 10 mM 2OG + 200 μM isocitrate; red trace: 10 mM each: 2OG, isocitrate. (B) (FNR+R132H)@ITO/PGE electrode, 5 μM NADP^+^; 20 mM each: MES, TAPS, CHES; stationary electrode. Purple trace: 10 mM each: 2OG, isocitrate. Enzyme loading ratios (molar): (A) FNR/IDH1/CA; 1/0.5/0.25; (B) FNR/R132H; 1/2.5.

Since the overall production of 2HG from isocitrate occurs without net consumption of NADP^+^, an experiment was conducted to determine the ability of R132H to catalyze isocitrate oxidation to 2OG under the special condition (approached through nanoconfinement) that NADPH formed and released from the enzyme is rapidly reoxidized by FNR (Figure 2B). The presence of 10 mM isocitrate at pH 8 resulted in a small increase in oxidation current positive of the NADP^+^/NADPH potential; subsequent addition of an equal amount of 2OG then produced a much larger reduction current negative of the NADP^+^/NADPH potential. The rate of isocitrate oxidation catalyzed by R132H (without 2OG present) was ∼18% that of 2OG reduction. The result confirms that R132H catalyzes production of 2OG from isocitrate as well as its subsequent reduction to 2HG. Determining the isocitrate oxidation activity of IDH1 variants^4,25^ is otherwise complicated because the NADPH product (determined spectrophotometrically) can be consumed by the competing 2OG/2HG reduction reaction.

To assess the overall thermodynamic landscape governing the appearance of 2HG in cells in which a neomorphic IDH variant is present, a Pourbaix (potential/pH) diagram (Figure 3) was constructed for the pH range 6–9 using the 2OG/isocitrate formal potential calculated from Figure 2A and a formal potential of −0.177 V vs SHE at pH 7 for the 2OG/2HG couple calculated from published data.^26,27^ Slopes were estimated assuming a 2e^–^/2H^+^ ratio (−60 mV/pH slope) for the 2OG/2HG couple and a 2e^–^/1H^+^ ratio (−30 mV/pH slope) for the 2OG/isocitrate and NADP^+^/NADPH couples.

**Figure 3:**
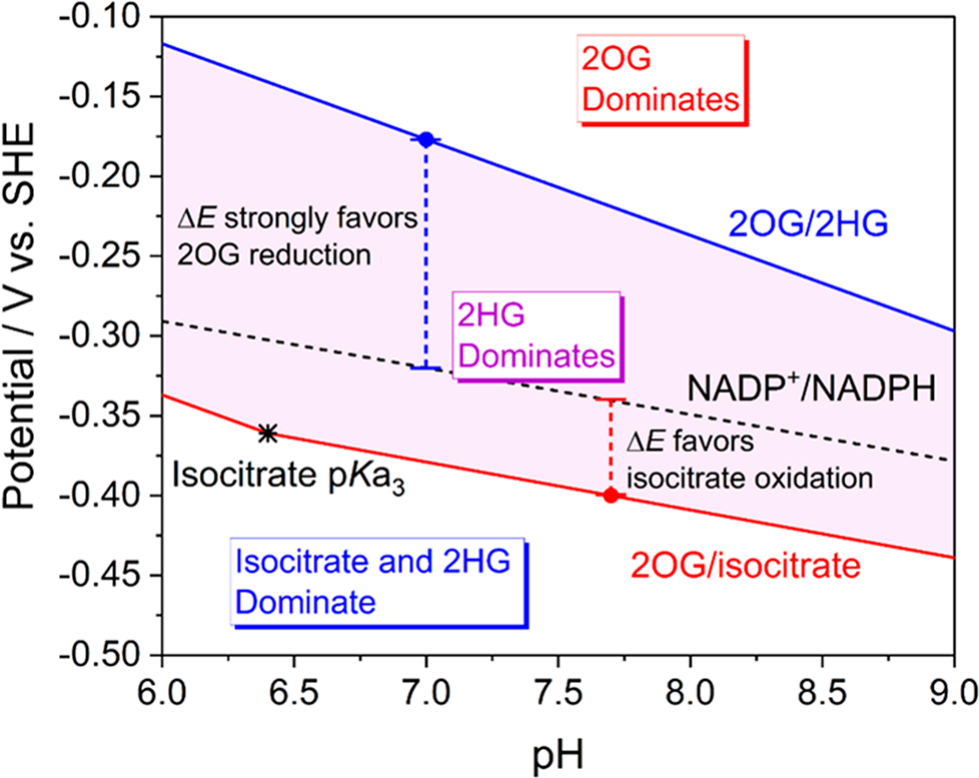
Pourbaix diagram depicting species stable in different regions of potential and pH. The central shaded region shows conditions where isocitrate will be spontaneously converted to 2HG. The slope of the 2OG/isocitrate reaction increases to −60 mV/pH below pH 6.4 as it becomes a 2e^–^/2H^+^ reaction: the third isocitrate carboxylate group becomes protonated whereas 2OG is a dianion above pH 4.8. The Δ*E* values show the bias for 2OG reduction or isocitrate oxidation mediated by NADP^+^/NADPH cycling.

The Pourbaix diagram displays the ranges of thermodynamic stability for the different metabolites, with the purple shaded region representing the potential/pH conditions under which isocitrate will be converted spontaneously into 2HG in a redox-neutral reaction where 2OG and NADPH are intermediates (the biologically relevant free energy change is approximately –40 kJ/mol at pH 7 based on a potential difference of –0.18 – (–0.38) = +0.20 V). Based on a typical potential for living cells being more negative than −0.2 V at neutral pH,^28^ the results imply that 2HG should accumulate whenever 2OG and/or isocitrate are being generated and a neomorphic IDH variant is present. Generally, the kinetic ability of dehydrogenases to couple metabolite interconversion with NADP(H) and the bias displayed for a particular direction depend on the gap (Δ*E*) between metabolite and NADP^+^/NADPH potentials.

The results presented so far reaffirm the ease with which enzymes energized by nanoconfined NADP^+^/NADPH recycling can function with electrochemically switchable bidirectionality, a property exploited recently to design a deracemizer for secondary alcohols.^23^ We reasoned that with such tight coupling, the direct relationship between enzyme rate and current would permit direct, real-time measurements of the kinetics of binding and release of inhibitors.

First, we investigated how quickly nanoconfined IDH1 and R132H respond to rapid changes in the external concentrations of their normal substrates and Mg^2+^ (the essential metal cofactor). Figure 4A shows the rates of development of isocitrate oxidation (IDH1) or 2OG reduction (R132H) initiated by injecting Mg^2+^ or respective substrates. In all cases, >75% activity is gained within seconds, followed by a slower growth that takes several minutes to plateau. When EDTA is added equimolar with Mg^2+^, the activity drops to a very low level after a few minutes but is rapidly restored upon further injection of Mg^2+^ (Figure 4B).

**Figure 4:**
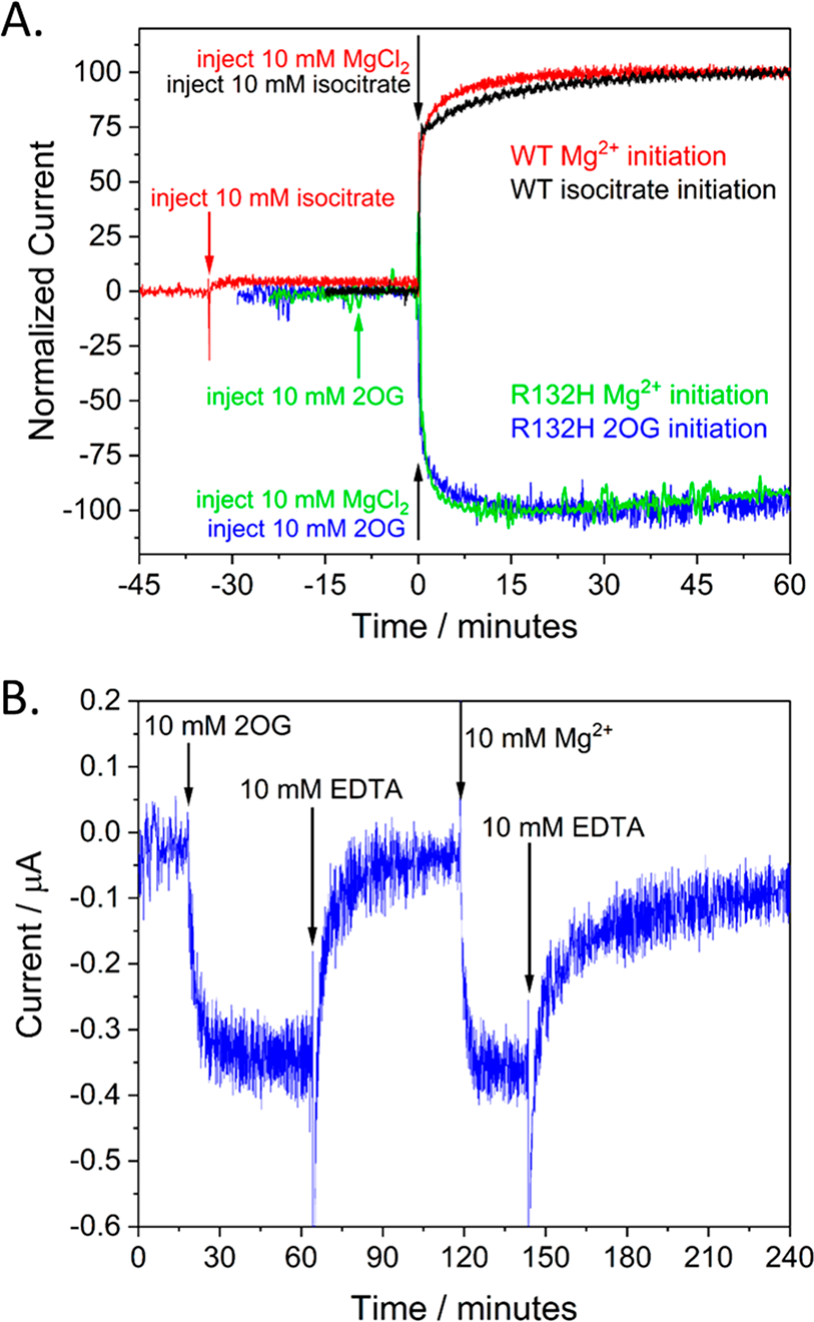
(A) Time courses for catalysis by IDH1 and R132H following injections of substrates and Mg^2+^. (B) Time course for inactivation/reactivation of R132H by removal of initial Mg^2+^ with EDTA, readdition of Mg^2+^, and then removal again by EDTA. Conditions: (FNR+E2)@ITO/PGE electrode, area 0.06 cm^2^, rotated at 1000 rpm, temperature 25 °C, volume 4 mL, potential *E* (vs SHE) = −0.513 V for R132H and −0.188 V for IDH1, pH = 8 (20 mM each: MES, TAPS, CHES), 10 mM MgCl_2_ (for experiments not initiated with Mg^2+^). 10 μM NADPH for R132H; 10 μM NADP^+^ for IDH1. Enzyme loading ratios (molar): FNR/IDH1; 1/0.5 and FNR/R132H; 1/2.5.

Having established that nanoconfined IDH1 and R132H can be activated or inactivated reversibly and the responses easily monitored, attention was directed at clinically relevant inhibitors of R132H (Figure 5). Panels A and B show the effect on R132H activity upon introducing different concentrations of Ivosidenib (AG-120) or Novartis 224 (Nov224) (as concentrated DMSO solutions) compared to DMSO-only controls. These inhibitors are known to react slowly,^2,10^ so the current was monitored for >3 h while the electrode was rotated at 1000 rpm to assist transport from solution. With the highest inhibitor concentrations (1 and 5 μM), R132H activity began to decrease immediately after injection. At lower concentrations (50 and 100 nM), R132H activity decreased more slowly, but still at a rate significantly above the control range (shaded region, Figure 5). At the lower concentrations, the inhibitors could be efficiently removed by a simple solution exchange once a substantial degree of inactivation had occurred. After removing the inhibitor, activity quickly started to recover, demonstrating that the inhibition of nanoconfined R132H can be reversed. A further control using Enasidenib (AG-221), an inhibitor specific for mitochondrial IDH2 variants,^29^ was carried out. The very slow decrease in current, just above the control threshold, is compelling evidence that the results with AG-120 and Nov224 reflect specific enzyme–inhibitor interactions and not physical interactions between the inhibitors and the system.

**Figure 5:**
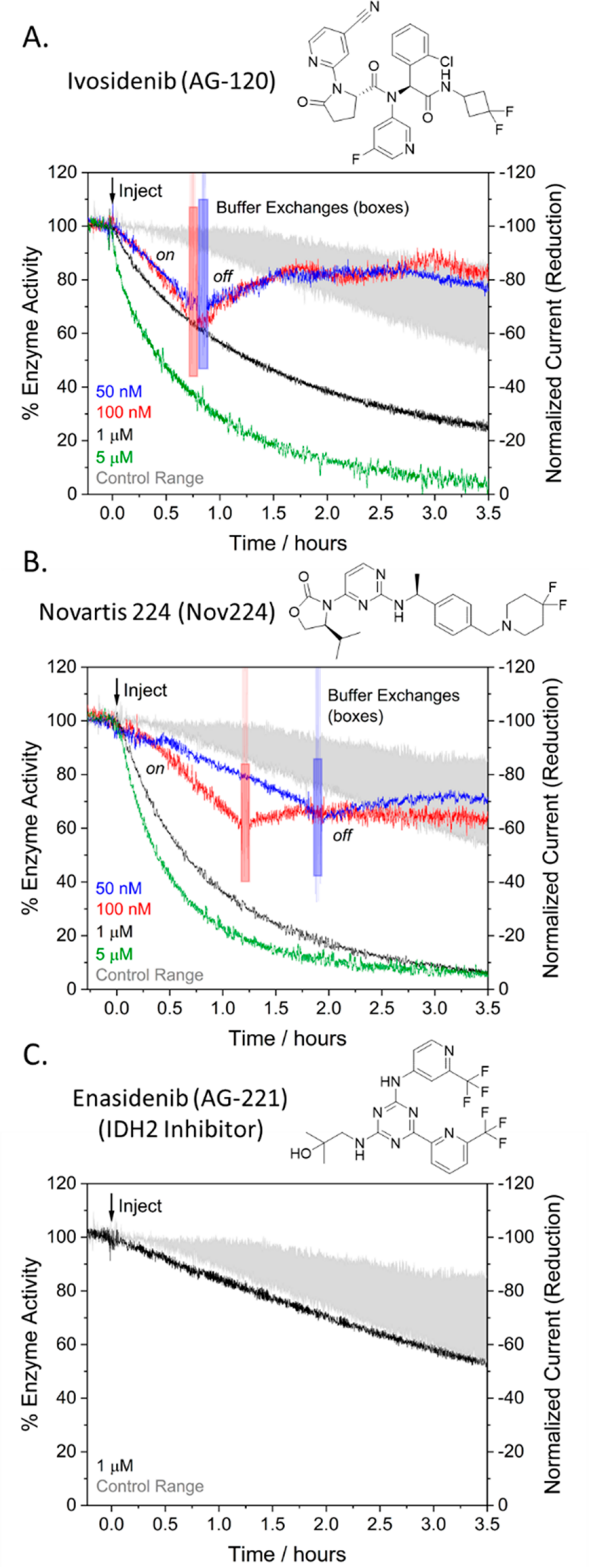
Effects on R132H activity by introducing Ivosidenib (AG-120) (A) or Novartis 224 (Nov224) (B) and then removing inhibitor (50 and 100 nM) by solution exchange (see the Supporting Information). (C) Control using Enasidenib (AG-221), specific for IDH2 variants. Conditions: (FNR+R132H)@ITO/PGE electrode, area 0.06 cm^2^, temperature 25 °C, *E* = −0.513 V vs SHE, mixed buffer (pH = 8): 20 mM each: MES, TAPS, CHES, 10 mM MgCl_2_, 10 μM NADPH, 10 mM 2OG, volume: 4 mL. Enzyme loading ratios (molar): FNR/R132H; 1/2.5. Inhibitor (2.5 μL of various concentrations in DMSO) was injected at *t* = 0. The shaded region shows the range for 3 DMSO controls.

The in situ, time-resolved kinetic data on the R132H–drug interaction provide an alternative and more mechanistically useful approach to complement the conventional IC_50_ (concentration required to decrease enzyme activity by 50%) metric typically used to assess inhibitors: the advantage is particularly relevant when dealing with drugs that react slowly with their target enzymes. Whereas IC_50_ is a useful empirical measure of inhibitor potency, it gives no direct information about inhibition kinetics, particularly under turnover conditions. Furthermore, because IDH1 inhibition is typically slow, an incubation period is required after adding inhibitor to the enzyme solution before adding substrate and measuring the initial reaction rate; the IC_50_ is therefore time dependent.^29,30^ For this work, IC_50_ values of 3.4 ± 0.3 and 4.2 ± 0.3 nM (±standard error) were measured for AG-120 and Nov224, respectively, following a 12 min incubation with IDH1 R132H (see the Supporting Information): the corresponding reported values for AG-221 are 78 μM after 1 h and 48 μM after 16 h of incubation.^29^

Classical dilute solution assays, including with varied incubation times, provide information about binding affinities and (sometimes) *on* and *off* rate constants. Information on the latter can also be obtained by biophysical techniques such as surface plasmon resonance (SPR), however, not under turnover conditions.^31^ Measuring the time-dependence of inhibition under realistic turnover conditions is challenging but important because (co)substrate/product binding/release often involves conformational changes that can affect inhibitor binding and hence potency. This aspect is of particular importance with structurally dynamic oligomeric enzymes such as IDH which are kinetically complex.^32^ In contrast to existing methods, the Electrochemical Leaf (Scheme 1) enables efficient direct measurements of the *rates* of action of inhibitors, both *on* and *off*, during a single experiment under turnover conditions (Figure 5). This advantage may be crucial in predicting how an inhibitor may behave *in vivo* where the target enzyme is processing substrate(s), especially since many IDH1 variant inhibitors are known to inhibit competitively to varying degrees with 2OG^13^ and Mg^2+^.^30^

An example of how the new information may be used is as follows: from the limited data set so far obtained, it appears that for both AG-120 and Nov224, the rate of inhibition approaches similar limiting values at a high concentration of the inhibitor, with the half-life of 20–30 min at 5 μM inhibitor equating to apparent first-order rate constants in the region of 4–6 × 10^–4^ s^–1^. Inspection of the *off* rates indicates a similar time scale, suggesting that both *on* and *off* rates may be determined by a common intramolecular process occurring in IDH1 R132H. For drug research, such an approach should greatly simplify the process of determining the kinetics and equilibria associated with inhibitor binding.

More widely, the Electrochemical Leaf approach may eventually provide interesting comparisons of enzyme catalysis and inhibition under open dilute vs nanoconfined and locally concentrated conditions—the latter of which may more accurately reflect conditions in living cells.^33^

## Supporting Information Available

Materials and Methods: Chemicals and reagents, enzyme expression and purification, electrode fabrication, enzyme loading method, electrochemical quantification of FNR, electrochemical measurement details, live buffer exchange protocol, enzyme solution assays and IC_50_ measurement, data smoothing. Supporting Results: Figure S1: Cyclic voltammograms (pH 6–9) for IDH1 and R132H. Figure S2: Spectrophotometric solution assays (pH 6–9) for IDH1 and R132H. Figure S3: Cyclic voltammetry using an electrode loaded with equimolar IDH1 and R132H. Figure S4: Stationary cyclic voltammetry showing IDH1 performing reductive carboxylation of 2OG. Figure S5: Chronoamperometry for R132H with 2OG titrated into solution. Figure S6: Exemplar data for a typical R132H inhibition experiment before normalization and data smoothing. Figure S7: DMSO controls for inhibition experiments. Figure S8: Repeat R132H inhibition experiments using Ivosidenib. Figure S9: Repeat R132H inhibition experiments using Novartis 224. Figure S10: Repeat R132H control experiment using Enasidenib (PDF)

## Abbreviations

FNR: ferredoxin-NADP^+^ reductase
NADP(H): nicotinamide adenine dinucleotide phosphate
IDH: isocitrate dehydrogenase
ITO: indium tin oxide
AML: acute myeloid leukemia
FAD: flavin adenine dinucleotide
CA: carbonic anhydrase.
