# Supplementary material for: Exploiting Electrode Nanoconfinement to Investigate the Catalytic Properties of Isocitrate Dehydrogenase (IDH1) and a Cancer-Associated Variant
Source: J Phys Chem Lett. 2021 Jun 25;12(26):6095–101. doi: 10.1021/acs.jpclett.1c01517 (PMC8273889; doi:10.1021/acs.jpclett.1c01517)
Supplement: Supplementary file 1 — jz1c01517_si_001.pdf [file jz1c01517_si_001.pdf]

Supporting Information

Exploiting Electrode Nanoconfinement to Investigate the Catalytic Properties of Isocitrate Dehydrogenase (IDH1) and a Cancer-associated Variant

Ryan A. Herold, Raphael Reinbold, Clare F. Megarity, Martine I. Abboud,†
Christopher J. Schofield,* and Fraser A. Armstrong*

Department of Chemistry, University of Oxford, Oxford OX1 3QR, United Kingdom

†Present Address: Department of Natural Sciences, Lebanese American University, Byblos/Beirut, Lebanon

Materials and Methods

Chemicals and Reagents

NADPH (tetrasodium salt, 93%, Melford), $NADP^+$ (monosodium salt, 98%, Melford), indium tin oxide (ITO) powder (< 50 nm particle size, Sigma-Aldrich), acetone (ACS Reagent), [2-(N-Morpholino)-ethanesulfonic acid] (MES) (monohydrate, Melford), [tris(hydroxymethyl)methylamino]-propanesulfonic acid (TAPS) (99%, Melford), [2-(N-Cyclohexylamino)ethanesulfonic acid] (CHES) (>99%, Melford), [N-(2-Hydroxyethyl) piperazine N'-(2-ethanesulfonic acid)] (HEPES) (free acid, Melford), α-ketoglutaric acid (disodium salt dihydrate, ≥98%, Sigma-Aldrich), DL-isocitric acid (trisodium salt hydrate, ≥93%, Sigma-Aldrich), ethylenediaminetetraacetic acid (EDTA) (disodium salt dihydrate, 99–101% (titration), Sigma-Aldrich), sodium bicarbonate (ACS Reagent, ≥99.7%, Sigma-Aldrich), $MgCl_2$ (anhydrous, Melford), carbonic anhydrase (from bovine erythrocytes) (lyophilized powder, >95%, Sigma-Aldrich), iodine (resublimed crystals, 99.9985% (metals basis), Puratronic, Alfa Aesar), Isopropyl β-D-1-thiogalactopyranoside (IPTG) (Alfa Aesar). All aqueous solutions were prepared using ultrapure water (≥18.2 MΩ cm, Milli-Q).

Inhibitors: AG-120 (Cat. No.: HY-18767, 99.78%), Nov224 (Cat. No.: HY-18717, >98.0%), and AG-221 (Cat. No.: HY-18690, 99.93%) inhibitors were purchased from MedChemExpress and were dissolved in DMSO (10 mM) and stored at -20 °C.

Expression and Purification of Recombinant Ferredoxin $NADP^+$-reductase

Ferredoxin-$NADP^+$ reductase (FNR) from *Chlamydomonas reinhardtii* was expressed in *E. coli* and purified as previously described.[1]

Expression and Purification of Recombinant *Hs* IDH1 and *Hs* IDH1 R132H

DNA encoding for human wild-type or R132H IDH1 with a 6XHis-tag at their C-terminus was inserted into the pET-22b(+) vector transformed into *Escherichia coli* BL21(DE3) pLyS cells, using 2TY medium supplemented with 50 µg/mL ampicillin and 34 µg/mL chloramphenicol. Cells were grown at 37 °C until they reached OD_{600} = 0.6–0.8. Expression was induced with 1 mM IPTG at 18 °C for 12 hours. After centrifugation [Beckman-Coulter, J-Lite® JLA-10.500, 500 mL centrifuge tubes, 11,876 g (8,000 rpm), 10 min. cycles, 6°C], cells were resuspended in 50 mL of lysis buffer (20 mM Tris-HCl, 500 mM NaCl, pH 7.4) supplemented with DNase I, TCEP, lysozyme and a tablet of EDTA-free protease inhibitors (Merck). Cells were then lysed by sonication (Cole-Parmer, 60 % amplitude, 9.9 s on, 9.9 s off) on ice. After centrifugation [Beckman-Coulter, JA-25.50, 50 mL tubes, 69,486 g (24,000

rpm), 30 min cycles, 6°C], cell lysates were loaded onto a 5 mL HisTrap HP column (GE Healthcare Life Sciences, Little Chalfont, UK), with 50 mM Tris-HCl, 500 mM NaCl, pH 7.4, containing 20 mM imidazole, then eluted with an imidazole gradient (up to 500 mM imidazole). Fractions containing recombinant IDH1 were further purified using a Superdex S200 column (300 mL) equilibrated with 20 mM Tris-HCl, pH 7.4, 100 mM NaCl. Fractions containing the purified protein were concentrated by centrifugal ultra-filtration. The *apo*-proteins were generated by overnight treatment at 4 °C with ethylenediaminetetraacetic acid (EDTA) (2000x molar excess of protein), followed by purification by PD-10 desalting column. The purity of the resulting proteins was assessed as >95% (by SDS-PAGE) and their concentrations were determined using a ND-1000 NanoDrop spectrophotometer.

ITO/PGE Electrode Fabrication

Nanoporous indium tin oxide (ITO) electrodes were prepared by electrophoretic deposition of ITO nanoparticles (<50 nm particle size, Sigma-Aldrich) onto pyrolytic graphite edge (PGE) rotating disc electrodes. PGE rotating disc electrodes were prepared in-house as previously described.[2] ITO nanoparticles were deposited from a suspension of ITO (0.02 g) with I_2 (0.01 g) dissolved in acetone (20 mL) that had been sonicated for 1–2 hours. The electrode and a conductive support were held in parallel orientation in the ITO suspension (approximately 1 cm apart), and a voltage of 10 V was applied (zero current) for 6 minutes with the PGE electrode connected to the negative terminal. After six minutes, the ITO electrode was allowed to dry and was visually inspected. If the nanoporous ITO layer appeared thin (more blue/green than green/yellow), it was placed back in the solution with the same voltage applied for up to an additional two minutes. The electrode was rinsed with water before use.

Enzyme Loading Method

Enzymes were loaded onto the electrode by dropcasting a concentrated 4–7 µL solution onto the ITO electrode and allowing it to incubate at room temperature >30 minutes while ensuring the solution did not evaporate. In all cases, 0.85 nmol (homodimer basis) of IDH1 (WT or R132H) was used and the corresponding amounts of FNR and carbonic anhydrase were adjusted to give the desired enzyme ratios. As an example, a 4 µL mixed enzyme solution was loaded on the electrode for all inhibition experiments that consisted of 1 µL of 0.34 mM FNR + 3 µL of 0.28 mM IDH1 R132H (giving a FNR/R132H; 1/2.5 molar ratio). Molar concentrations for the IDH1 enzymes were calculated based on their respective homodimers. Electrodes were rinsed thoroughly using buffer before use in an experiment to ensure that no enzyme was introduced into the cell solution.

Electrochemical Quantification of FNR Coverage

Electroactive FNR adsorbed on the electrode was quantified by integrating and averaging the FNR-bound FAD redox peaks ("non-turnover" peaks) from cyclic voltammograms and using the equation

$$\Gamma = \frac{\text{Peak Area}}{nFA\upsilon}$$

where Γ is the amount (in moles) of electroactive FNR per unit area (mol/cm^2), peak area is the average area of the integrated FNR-bound FAD reduction and oxidation peaks (in units AV or J/s), υ is the scan rate (V/s), F is the Faraday constant (96485 C/mole), A is the electrode surface area (cm^2), and n is the number of electrons transferred (2 electrons per molecule of FNR).

Electrochemical Measurements

All electrochemical experiments were carried out in an anaerobic glove box (Glove Box Technology Limited) with a nitrogen atmosphere (O_2 < 1 ppm). Cyclic voltammetry and chronoamperometry experiments were carried out using an Autolab PGSTAT 10 potentiostat using Nova software. Experiments were carried out using an in-house glass electrochemical cell as previously described,[2]

with the main cell compartment surrounded by a water jacket to control temperature. The reference electrode was housed in a non-isothermal side arm containing 0.10 M NaCl and connected to the main cell compartment by a Luggin capillary. Electrode potentials (E) were measured against a saturated calomel electrode (SCE) and converted to the standard hydrogen electrode (SHE). To correct for temperature variation (20-27 °C) inside the glovebox (which determined the temperature of the reference electrode), the appropriate conversion factor was used from **Table S1**. The temperature-dependent conversion values given in the table were calculated from an equation obtained from Bard and Faulkner, 1980.[3] The equation used to convert SCE to SHE was: $E_{SHE} = E_{SCE}$ + Correction (V) (at 25 °C the equation is: $E_{SHE} = E_{SCE} + 0.2412$ V).

Table S1. Conversion of SCE to SHE Based on Reference Electrode Temperature

**Temperature (°C)**	Correction from SCE to SHE (V)	**Temperature (°C)**	Correction from SCE to SHE (V)
**15**	0.2476	**26**	0.2405
**16**	0.247	**27**	0.2399
**17**	0.2464	**28**	0.2392
**18**	0.2457	**29**	0.2385
**19**	0.2451	**30**	0.2379
**20**	0.2445	**31**	0.2372
**21**	0.2438	**32**	0.2365
**22**	0.2432	**33**	0.2358
**23**	0.2425	**34**	0.2351
**24**	0.2419	**35**	0.2344
**25**	0.2412		

Live Buffer Exchange Protocol

The reaction buffer solution was exchanged using the same stock of buffer solution that was used at the start of each experiment (this time with 2OG already present—since it was used to initiate the reaction prior to inhibition), so that, apart from the absence of inhibitor, it was identical to the cell solution (pH = 8 (20 mM each: MES, TAPS, CHES), 10 mM $MgCl_2$, 10 µM NADPH, 10 mM 2OG). To exchange the reaction solution live during the experiment, a 1 mL syringe was used to remove ~1.5 mL of buffer (reducing 4 mL starting volume to ~2.5 mL) while ensuring the rotating electrode remained submerged in order to maintain electrical contact. A 60 mL syringe containing 35 mL of fresh buffer without inhibitor was then used to slowly inject 10 mL of buffer into the cell. The syringe was removed and a 20 mL syringe was then used to remove 10 mL of buffer from the cell now containing diluted inhibitor. A fresh aliquot of 10 mL of buffer was again added to the cell, and the process was repeated until all of the fresh buffer had been used and the final cell volume was reduced back to the starting 4 mL.

Enzyme Solution Assays and IC_{50} Measurement

Spectrophotometric assays

Assays were conducted in a buffer containing 100 mM Tris base, 10 mM $MgCl_2$, 0.2 mM DTT, Tween-20 (0.005 % (C_V)), and 0.1 mg/mL BSA. The pH was adjusted to 6, 7, 8, or 9 using HCl or NaOH. Wild-type *Hs* IDH1 (2.5 nM) was allowed to warm up to room temperature, and the reaction was

initiated by addition of 0.3 mM isocitrate and 0.1 mM $NADP^+$. *Hs* IDH1 R132H (200 nM) was allowed to warm up to room temperature, and the reaction was initiated by addition of 1.5 mM 2OG and 0.3 mM NADPH. The turnover of NADPH was observed by measuring the absorbance at 340 nm using a PHERAstar FS Microplate Reader. Activities were normalised to pH 8 for each enzyme. IDH1 molar enzyme concentrations were calculated based on their respective homodimers.

IC_{50} Measurement

IC_{50} values were measured at pH 8 in a buffer containing 100 mM Tris base, 10 mM $MgCl_2$, 0.2 mM DTT, Tween-20 (0.005 % (C_V)), and 0.1 mg/mL BSA. *Hs* IDH1 R132H (15 nM) was incubated with inhibitor for 12 minutes before the reaction was initiated by addition of 1.5 mM 2OG and 0.3 mM NADPH. IC_{50} values were calculated using non-linear regression (GraphPad Prism). All experiments were conducted in triplicate. The molar enzyme concentration for IDH1 R132H was calculated based on the R132H homodimer.

Data Smoothing

Some R132H chronoamperometry traces were smoothed using the Nova software Savitzky-Golay method (polynomial order 2, 4 points left/right). R132H traces in the main text that were smoothed are: **Figure 3A** and **Figure 4A-C**. See **Figure S6** for an example of an R132H trace before and after smoothing.

Supporting Results

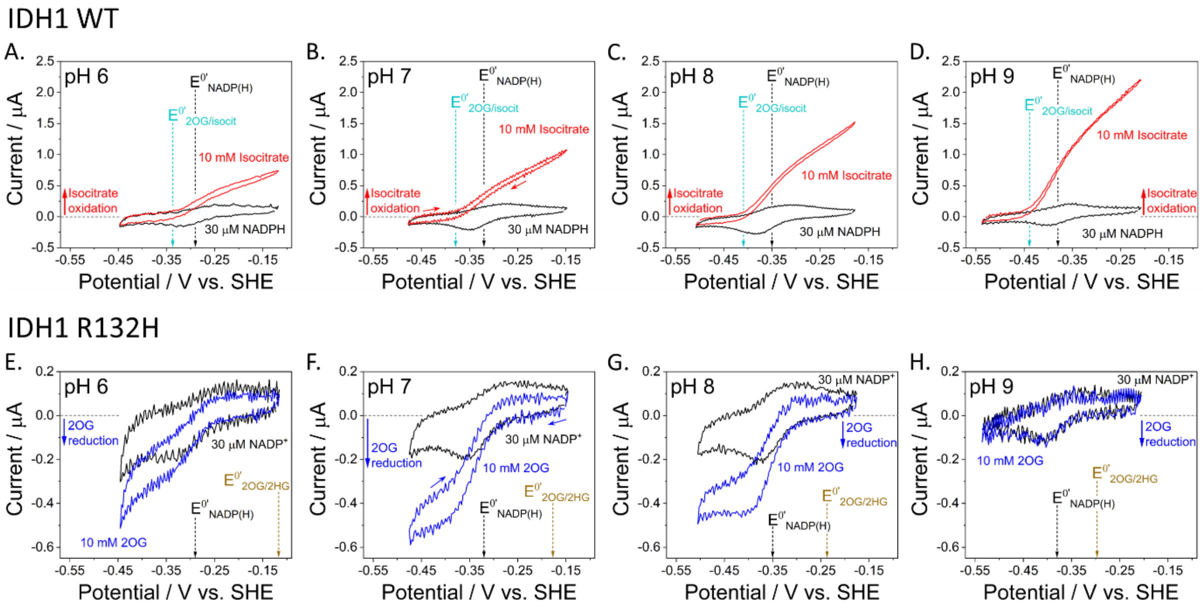

Figure S1. Cyclic voltammograms for catalysis by IDH1 (**A**–**D**) and IDH1 R132H (**E**–**H**) at different pH (pH 7 and 8 for both enzymes are presented in **Figure 1** in the main text). Conditions: stationary (FNR+E2)@ITO/PGE electrode, electrode area 0.03 cm^2, scan rate 1 mV/s, temperature 25 °C, mixed buffer (pH = 8): 20 mM each: MES, TAPS, CHES, 10 mM $MgCl_2$, 30 μM NADP(H), 10 mM substrate (isocitrate or 2-oxoglutarate), volume: 4 mL. Enzyme loading ratios (molar basis): FNR/IDH1; 1/0.5, FNR/R132H; 1/2. Markers $E^{0'}_{NADP(H)}$, $E^{0'}_{2OG/isocit}$, and $E^{0'}_{2OG/2HG}$ denote formal potentials for $NADP^+$/NADPH, 2OG/isocitrate, and 2OG/2HG couples.

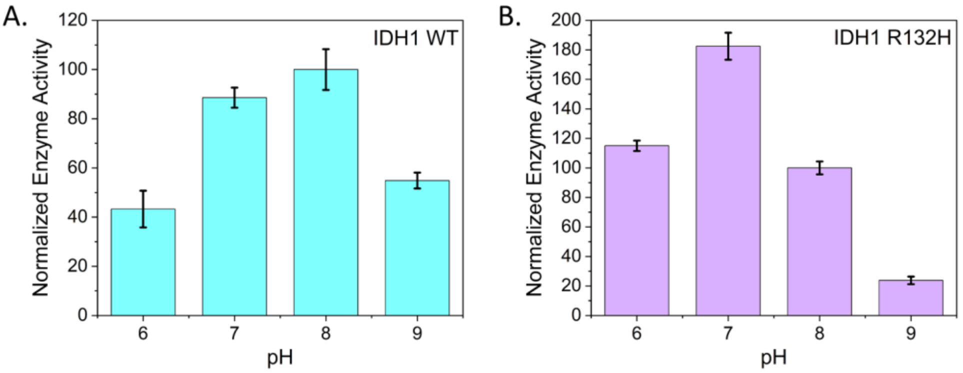

Figure S2. Spectrophotometric solution assays showing the effect of pH on catalytic rate for wild-type IDH1 (**A**) and IDH1 R132H (**B**) coupled to $NADP^+$ and NADPH, respectively. Rates were normalized to the activity at pH 8 for each enzyme. Error bars represent the standard deviation of three technical replicates.

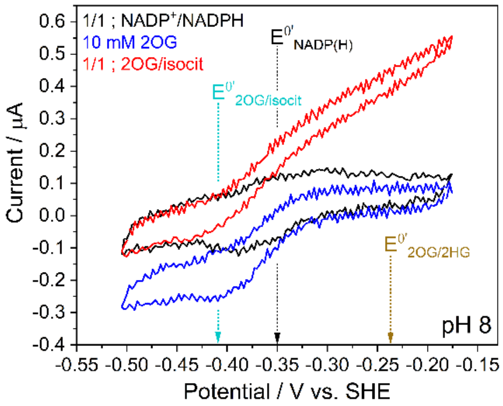

Figure S3. Cyclic voltammetry using an electrode loaded with equimolar wild-type IDH1 and IDH1 R132H demonstrating the difference in their respective catalytic turnover rates and the resulting characteristic shapes of their CV traces. Wild-type IDH1 exhibits a steep potential dependence for isocitrate oxidation (limited by the rate of $NADP^+$ recycling by FNR) while R132H exhibits a sigmoidal shaped trace for 2OG reduction with a current that does not increase as the applied overpotential is increased (R132H limited). Conditions: stationary (FNR+IDH1+R132H)@ITO/PGE electrode, electrode area 0.03 cm^2, scan rate 1mV/s, temperature 25 °C, cell volume: 4 mL, mixed buffer (pH = 8): 20 mM each: MES, TAPS, CHES, 10 mM $MgCl_2$, 10 µM $NADP^+$, 10 µM NADPH. Red trace: 10 mM 2OG + 10 mM isocitrate. Enzyme loading ratios: FNR/IDH1/R132H; 1/2/2 (0.85 nmol of each IDH1 enzyme was loaded).

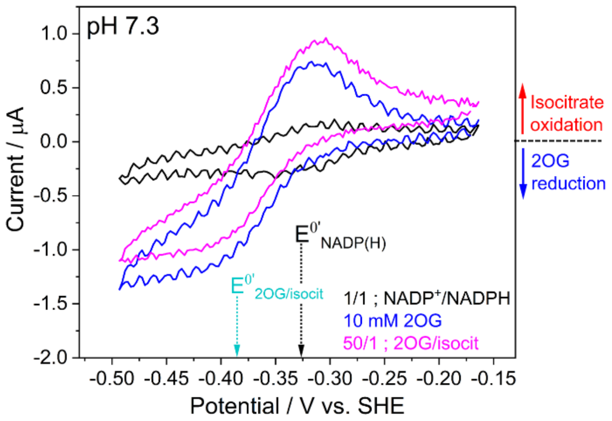

Figure S4. Cyclic voltammetry showing the strong preference of wild-type IDH1 for isocitrate oxidation over 2OG reduction (trace amounts of isocitrate formed by IDH1-catalyzed 2OG reduction at low potentials is reoxidized at more positive potentials at stationary electrode). Conditions: stationary (FNR+IDH1+CA)@ITO/PGE electrode, electrode area 0.06 cm^2, scan rate 1 mV/s, temperature 25 °C, cell volume: 4 mL, 10 mM $MgCl_2$, 15 µM $NADP^+$, 15 µM NADPH, HEPES and $NaHCO_3$ at 0.10 M. Magenta trace: 10 mM 2OG + 200 µM isocitrate. Carbonic anhydrase = CA. Enzyme loading ratios: FNR/IDH1/CA; 1/0.5/0.25.

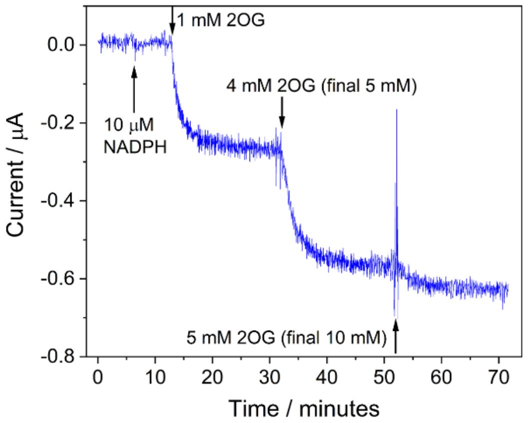

Figure S5. Increasing IDH1 R132H reduction activity as 2OG is titrated into solution. Conditions: (FNR+R132H)@ITO/PGE electrode, electrode area 0.06 cm^2, rotated at 1000 rpm, temperature 25 °C, potential *E* (vs. SHE) = -0.513 V, mixed buffer (pH = 8): 20 mM each: MES, TAPS, CHES, 10 mM $MgCl_2$, 10 µM NADPH. Enzyme loading ratios (molar): FNR/R132H; 1/2.5.

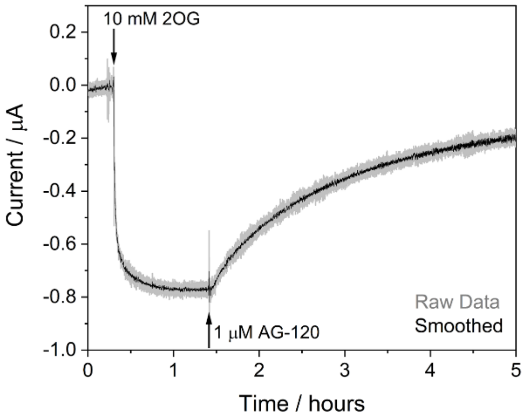

Figure S6. Example of raw data for a typical inhibition experiment before normalization (inverts current to positive values) and smoothing (gray trace) as well as after smoothing (black trace). Conditions: (FNR+R132H)@ITO/PGE electrode, electrode area 0.06 cm^2, rotated at 1000 rpm, temperature 25 °C, E = -0.513 V vs. SHE, mixed buffer (pH = 8): 20 mM each: MES, TAPS, CHES, 10 mM $MgCl_2$, 10 µM NADPH, 10 mM 2-oxoglutarate, volume: 4 mL. Enzyme loading ratios (molar): FNR/R132H; 1/2.5. AG-120 in DMSO (2.5 µL of 1.6 mM) was injected at the time indicated. Black trace was smoothed using the Savitzky-Golay method (4 points left/right).

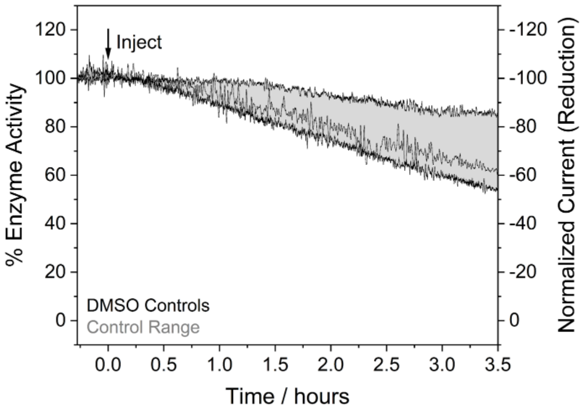

Figure S7. DMSO control chronoamperometry experiments. Conditions: (FNR+R132H)@ITO/PGE electrode, electrode area 0.06 cm^2, rotated at 1000 rpm, temperature 25 °C, E = -0.513 V vs. SHE, mixed buffer (pH = 8): 20 mM each: MES, TAPS, CHES, 10 mM $MgCl_2$, 10 µM NADPH, 10 mM 2-oxoglutarate, volume: 4 mL. Enzyme loading ratios (molar): FNR/R132H; 1/2.5. DMSO (2.5 µL) was injected at time = 0, with the shaded region representing the integrated area between control experiments. Traces were smoothed using the Savitzky-Golay method (4 points left/right).

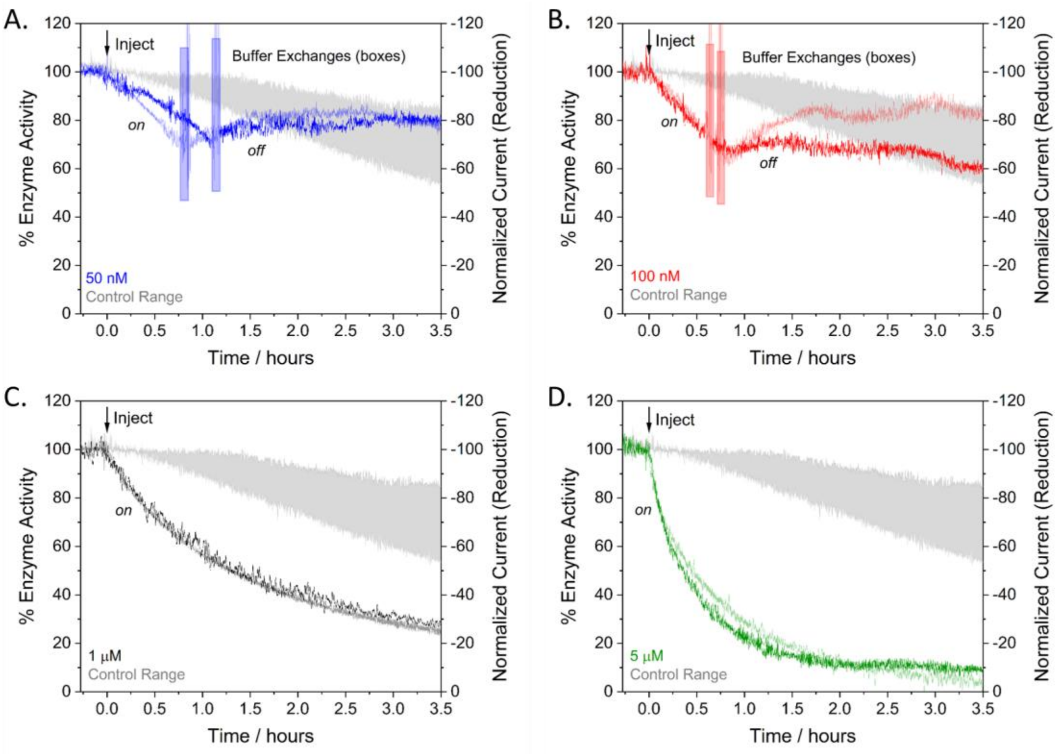

Figure S8. Repeat IDH1 R132H inhibition experiments using Ivosidenib (AG-120) for each condition used in the main text of the paper: 50 nM (**A**), 100 nM (**B**), 1 µM (**C**), and 5 µM (**D**). The faded traces are those presented in the main text and the darker traces are repeat experiments. Buffer exchanges were used to remove the inhibitor for experiments at 50 and 100 nM (**A** and **B**). Conditions: (FNR+R132H)@ITO/PGE electrode, electrode area 0.06 cm^2, rotated at 1000 rpm, temperature 25 °C, E = -0.513 V vs. SHE, mixed buffer (pH = 8): 20 mM each: MES, TAPS, CHES, 10 mM $MgCl_2$, 10 µM NADPH, 10 mM 2-oxoglutarate, volume: 4 mL. Enzyme loading ratios (molar): FNR/R132H; 1/2.5. Inhibitor (2.5 µL of varying concentrations in DMSO) was injected at time = 0. Pure DMSO (2.5 µL) was injected for controls, the shaded region representing the range of three control experiments. Traces were smoothed using the Savitzky-Golay method (4 points left/right).

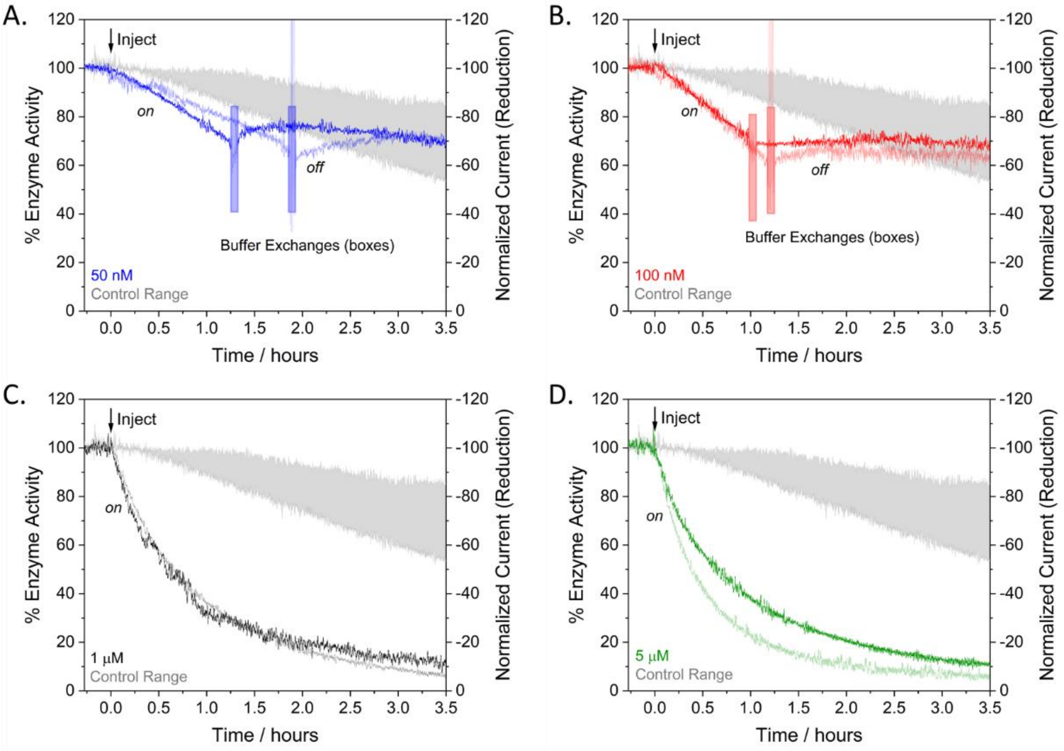

Figure S9. Repeat IDH1 R132H inhibition experiments using Novartis 224 (Nov224) for each condition used in the main text of the paper: 50 nM (**A**), 100 nM (**B**), 1 μM (**C**), and 5 μM (**D**). The faded traces are those presented in the main text of the paper and the darker traces are repeat experiments. Buffer exchanges were used to remove the inhibitor for experiments at 50 and 100 nM (**A** and **B**). Conditions: (FNR+R132H)@ITO/PGE electrode, electrode area 0.06 cm^2, rotated at 1000 rpm, temperature 25 °C, E = -0.513 V vs. SHE, mixed buffer (pH = 8): 20 mM each: MES, TAPS, CHES, 10 mM $MgCl_2$, 10 μM NADPH, 10 mM 2-oxoglutarate, 4 mL volume. Enzyme loading ratios (molar): FNR/R132H; 1/2.5. Inhibitor (2.5 μL of varying concentrations in DMSO) was injected at time = 0. DMSO (2.5 μL) was injected for controls, the shaded region representing the range of three control experiments. Traces were smoothed using the Savitzky-Golay method (4 points left/right).

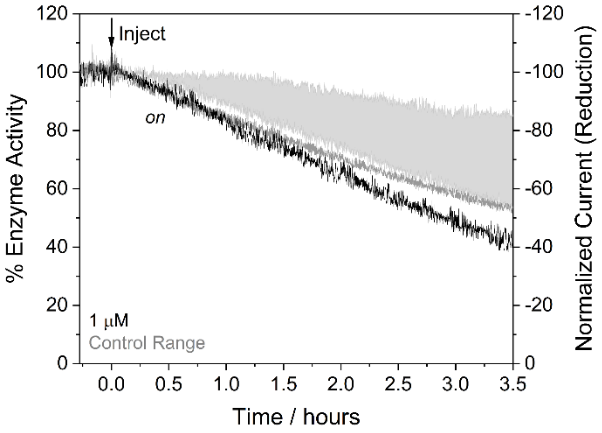

Figure S10. Repeat of control experiment using Enasidenib (AG-221), an inhibitor specific for mitochondrial IDH2 variants. The faded trace is presented in the main text of the paper and the darker trace is the repeat experiment. Conditions: (FNR+R132H)@ITO/PGE electrode, electrode area 0.06 cm^2, rotated at 1000 rpm, temperature 25 °C, E = -0.513 V vs. SHE, mixed buffer (pH = 8): 20 mM each: MES, TAPS, CHES, 10 mM $MgCl_2$, 10 μM NADPH, 10 mM 2-oxoglutarate, 4 mL volume. Enzyme loading ratios (molar): FNR/R132H; 1/2.5. Inhibitor (2.5 μL of 1.6 mM AG-221 in DMSO) was injected at time = 0. DMSO (2.5 μL) was injected for controls, with the shaded region representing the range of three control experiments. Traces were smoothed using the Savitzky-Golay method (4 points left/right).

References

(1) Megarity, C. F.; Siritanaratkul, B.; Heath, R. S.; Wan, L.; Morello, G.; FitzPatrick, S. R.; Booth, R. L.; Sills, A. J.; Robertson, A. W.; Warner, J. H.; Turner, N. J.; Armstrong, F. A. Electrocatalytic Volleyball: Rapid Nanoconfined Nicotinamide Cycling for Organic Synthesis in Electrode Pores. *Angew. Chemie Int. Ed.* **2019**, *58* (15), 4948–4952.

(2) Evans, R. M.; Armstrong, F. A. Electrochemistry of Metalloproteins: Protein Film Electrochemistry for the Study of E. Coli [NiFe]-Hydrogenase-1. In *Methods in Molecular Biology*; Humana Press Inc., 2014; Vol. 1122, pp 73–94.

(3) Bard, A. J.; Faulkner, L. R. *Electrochemical Methods: Fundamentals and Applications*, 1st ed.; John Wiley & Sons, 1980.
